# Student-led curriculum design and teaching by fellow students provide a feasible platform for surgical identity and a gateway to surgical careers

**DOI:** 10.1515/iss-2025-0014

**Published:** 2025-08-05

**Authors:** Hajera Khaleqi, Kira Meißner, Abdulrahman Al-Harazi, Kim Lea Seifert, Lara Čierná, Maxim Popov, Gaby Lee, Antonia Reers, Saman Keshtkaran, Pauline Schuppert, Florian Rapp, Lucas Cröpelin, Martin Oertel, Fabian Pascal Schönewald, Jenna Sophie Schöllhorn, Atiehalsadat Nasr Esfahani, Tobias Dust, Judith Stolz, Andreas Lindner, Anas Amin Preukschas, Felix Nickel, Amila Cizmic, Melanie Le, Michael Sennert, Konrad Reinshagen, Christian Tomuschat, Hans Christian Schmidt

**Affiliations:** SimLab – Students for Surgery, Student Research Group, University Medical Center, Hamburg-Eppendorf, Hamburg, Germany; Department of Pediatric Surgery, University Medical Center Hamburg-Eppendorf, Hamburg, Germany; Department of General, Visceral, and Thoracic Surgery, University Medical Center, Hamburg-Eppendorf, Hamburg, Germany; Department of Trauma and Orthopedic Surgery, University Medical Center, Hamburg-Eppendorf, Hamburg, Germany; Department of Pediatric Surgery, Childrens Hospital St. Marien gGmbH, Teaching Affiliate of the Ludwig-Maximilians University of Munich, Landshut, Germany; Department of Pediatric Surgery, Dr. Von Hauner Children’s Hospital, University Hospital, LMU Munich, Munich, Germany

**Keywords:** surgical training, surgical identification, peer-teaching

## Abstract

**Objectives:**

Simulation-based (SBT) training is an essential component of modern surgical education as it improves both technical and non-technical skills while increasing the motivation of medical students. This study investigates the implementation of a student-led SBT based on peer teaching to overcome challenges in surgical education, maintain motivation, and facilitate access to surgical professions.

**Methods:**

From January 2024 to December 2024 a student-led voluntary course on basic laparoscopic techniques was performed, in which medical students from all semesters could participate. Satisfaction, motivation, and perceived openness for surgical professions of the participants were assessed using questionnaires and evaluated descriptively.

**Results:**

Eighty six medical students were trained in basic laparoscopic skills. The main reasons for participating were the desire for practical training (80 %), interest in laparoscopic techniques (76 %), and the pursuit of a surgical career (56 %). Overall satisfaction was high (*M*=4.88/5). After the course, participants felt better prepared for clinical internships (87 %) and said they are more likely to pursue in the surgical field (74 %).

**Conclusions:**

Peer teaching in a non-institutionalized, exam-free environment shows considerable potential for promoting surgical identity in medical students. The results underscore the importance of voluntary, creative training formats to support motivation, competence development, and identity formation in surgery.

## Introduction

Simulation-based training (SBT) is considered a key method in medical curricula because it improves both technical and non-technical skills while increasing student engagement and confidence [1]. It provides a risk-free environment for skill acquisition, addressing ethical concerns by ensuring that foundational surgical competencies can be developed before performing procedures on patients [2], 3].

In the context of an unmet global surgical burden [4], an aging population and the expected increase in the number of surgical patients [5], [6], [7], the need to identify factors for recruiting new colleagues and to minimize turnover from surgical specialties intensifies [8]. Plus, medical students’ interest in surgical specialties is overall declining despite the attractiveness of these specialties [9]. Factors such as prevailing stereotypes, negative experiences, and missing role models are likely to deter students from taking an interest in surgical disciplines as early as their undergraduate studies. Creating positive role models, particularly through mentoring and peer-teaching, has been shown to be an important way of creating a positive trend to motivate initiatives to strengthen surgical identity and take up surgical careers [9], 10]. The student’s motivation could be supported by offering SBTs and considering the overall context and psychological resources of students and teachers during their studies [11], 12]. Although there is a lot of evidence for the added value of constructions for SBT simulation laboratories, it is surprising that this is not yet permanently available for all students in all training institutions.

This study shows an example of how independently peer-educators introduced surgical basics with support from experts to expand the range of surgical training opportunities. This exemplary setup of a training environment by students aims to address challenges in surgical education, maintaining motivation, and ensuring a gateway for surgeon recruitment and careers.

## Methods

To create a structured, engaging, and skill-enhancing environment for medical students, a student-led, peer-teaching laparoscopic training program was developed and conducted from January 2024 to December 2024. The primary objective was to enhance technical competence, foster long-term motivation for surgical training, and encourage participation in structured surgical education. The training focused on the acquisition of fundamental laparoscopic skills through a cascade training model. Basic exercises were carried out to familiarise participants with camera handling and working with both hands, with as little effort as possible, with the aim of achieving a basic level of proficiency in using both hands. Since cutting and suturing are particularly important in laparoscopy and are essential for surgical training, as well as teaching basic motor skills, spatial awareness, and instrumental dexterity, these were practised and finally completed with a classic laparoscopic suturing exercise, according to previous curricula [13], 14]. Medical students in higher semesters taught their fellow students in these exercises after being trained by surgical experts.

### Study design and implementation

Participation onto the course was offered to all medical students of the Faculty of Medicine at the University Hamburg. The curriculum was designed as a voluntary, extra-curricular course, with a 4 h structured training session per cohort. If the students agreed to participate in the study, a written informed consent form was signed. A 2:1 student-to-teacher ratio was maintained to ensure personalized instruction. Course dates were promoted via social media and semester-specific academic groups, and registration was facilitated through the university’s teaching platform (Mephisto). The availability of courses was adjusted based on tutor participation, ensuring a minimum of one session every two weeks, with additional sessions scheduled when more tutors were available.

### Training curriculum and teaching methodology

Participants engaged in a progressively structured training program, starting with introductory tasks and advancing to more complex laparoscopic skills. The curriculum was designed to accommodate varied learning preferences, allowing students to self-select modules, vary the original order and determine individual skill acquisition pace. The training followed a stepwise approach, transitioning from basic hand-eye coordination exercises to advanced suturing techniques.

The cascade training model was implemented as follows:

1. Initial instructor training: Surgical specialists trained a cohort of student tutors in ergonomics, communication, and laparoscopic techniques (e.g., suturing, instrument handling).
2. Peer-teaching: Trained tutors conducted the course, providing hands-on instruction while maintaining a structured learning environment.
3. Tutor recruitment: The most engaged participants were selected to become future tutors, establishing a self-sustaining, intergenerational peer-teaching system while ensuring consistency and high training standards.

During the course, tutors emphasized ergonomic principles, precise surgical communication, and real-time feedback. Participants were encouraged to take breaks as needed to maintain focus and minimize fatigue. Tutors provided structured guidance, addressing both technical and procedural questions.

### Training modules and learning stations

The training curriculum incorporated six predefined modules designed to develop laparoscopic dexterity, spatial awareness, and instrument precision:

1. Children’s Construction Model: Enhances camera handling, team communication, and basic coordination. Description: The first task helps the students to get to know each other and practice effective communication while developing the correct hand position with the camera. Person A is asked to hide five pegs named “metastasis” in the model, which person B then has to search for with the camera and display in full. Afterwards person A removes the peg using atraumatic laparoscopic grasping forceps. Then they switch. At the end of this exercise, each participant has found and removed a certain number of pegs, learned basic handling skills and built a foundation for communication before moving on to the next module.
2. Peg Transfer (Basic and Advanced): Develops precision, ambidexterity, and instrument control. Description: The exercises involved moving from a starting point to a target position in three variants. At the beginning, the pegs were transferred exclusively with the dominant hand, then with the non-dominant hand, and finally with both hands, whereby the pegs were transferred in the air to the other hand.
3. Pipe Cleaner Exercise: Improves bimanual dexterity and coordinated movements. Description: involves threading a pipe cleaner in an S-shaped manner through the rings in the air, helps participants improve their precision and coordination as they practice using both hands effectively.
4. Cutting Task: Introduces precision cutting using laparoscopic scissors. Description: In this task, participants are asked to cut out a figure along a given line.
5. Suturing Exercise: Builds foundational skills in laparoscopic suturing techniques. Description: The aim is to place an intracorporeal knot under supervision and adjust the wound edges using a silicone suture pattern.

Each workstation was equipped with box trainers, laparoscopic instruments, and standardized training modules. The setup allowed for interactive, hands-on learning, promoting skill acquisition in a structured yet low-pressure environment.

### Evaluation and data collection

Two self-designed questionnaires (Supplementary Materials 1 and 2) were used to assess the effectiveness of the training program. The evaluation focused on:

– Feasibility of training modules and students’ motivations for participating (Questionnaire 1).
– Self-perceived improvement, personal satisfaction, and motivation before and after the peer-teaching experience (Questionnaire 2).

Participants rated statements using a five-point Likert scale (1=strongly disagree, 3=neutral, 5=strongly agree).

### Data analysis

Survey responses were collected anonymously via QR-coded access to LimeSurvey (Version 6.6.6, LimeSurvey GmbH). Due to the limited sample size, quantitative analyses were restricted to descriptive statistics (IBM SPSS^®^ Version 29.0.2.0).

### Ethical considerations

The study did not constitute a “research project involving human beings” as defined in Section 9 (2) of the Hamburg Chamber Act for the Medical Professions and therefore did not require ethical consultation of the Ethics Committee of the Hamburg Medical Association (2023-300376-WF).

## Results

### Establishment of the laparoscopic training program

A total of 41 medical students participated in the initial phase of the laparoscopic training program (female: *n*=24, 59 %; male: *n*=17, 41 %; non-binary: *n*=0, 0 %). Most participants were in the clinical stage of their studies (*n*=27, 66 %), and over half had prior exposure to surgical environments (*n*=18, 59 %), primarily through assisting or observing procedures. However, fewer participants had prior experience with laparoscopic techniques (*n*=13, 32 %).

The primary motivations for enrollment were:

– Desire for hands-on training beyond the standard curriculum (*n*=33, 80 %),
– General interest in laparoscopic techniques (*n*=31, 76 %),
– Aspiration for a surgical career (*n*=24, 56 %).

**Figure 1:**
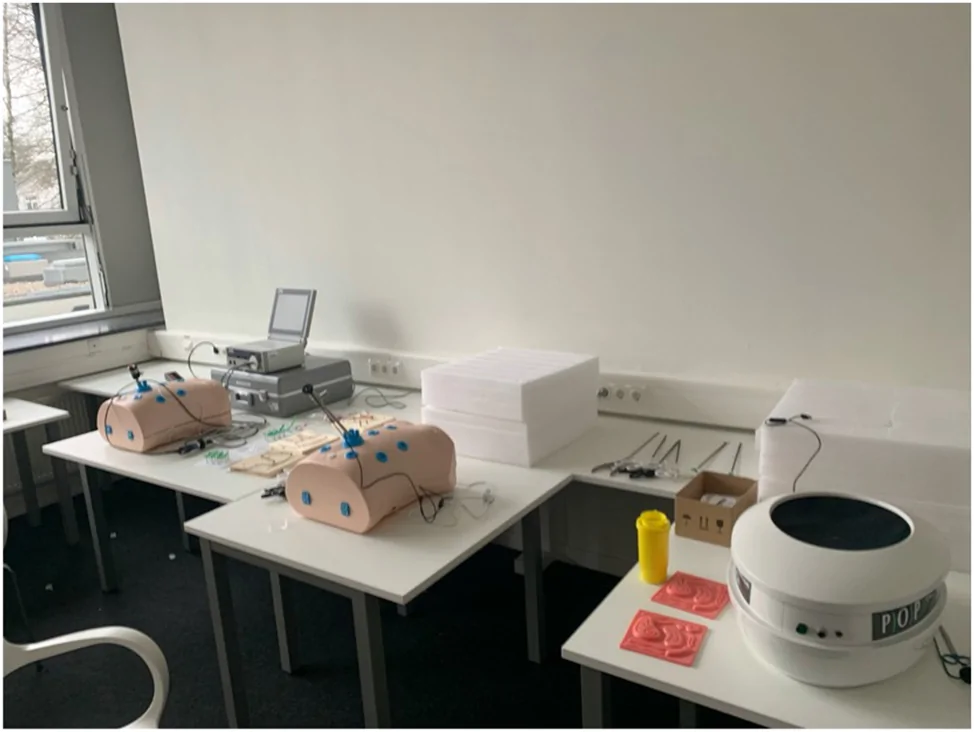
Training setting for the predefined curriculum.

**Figure 2:**
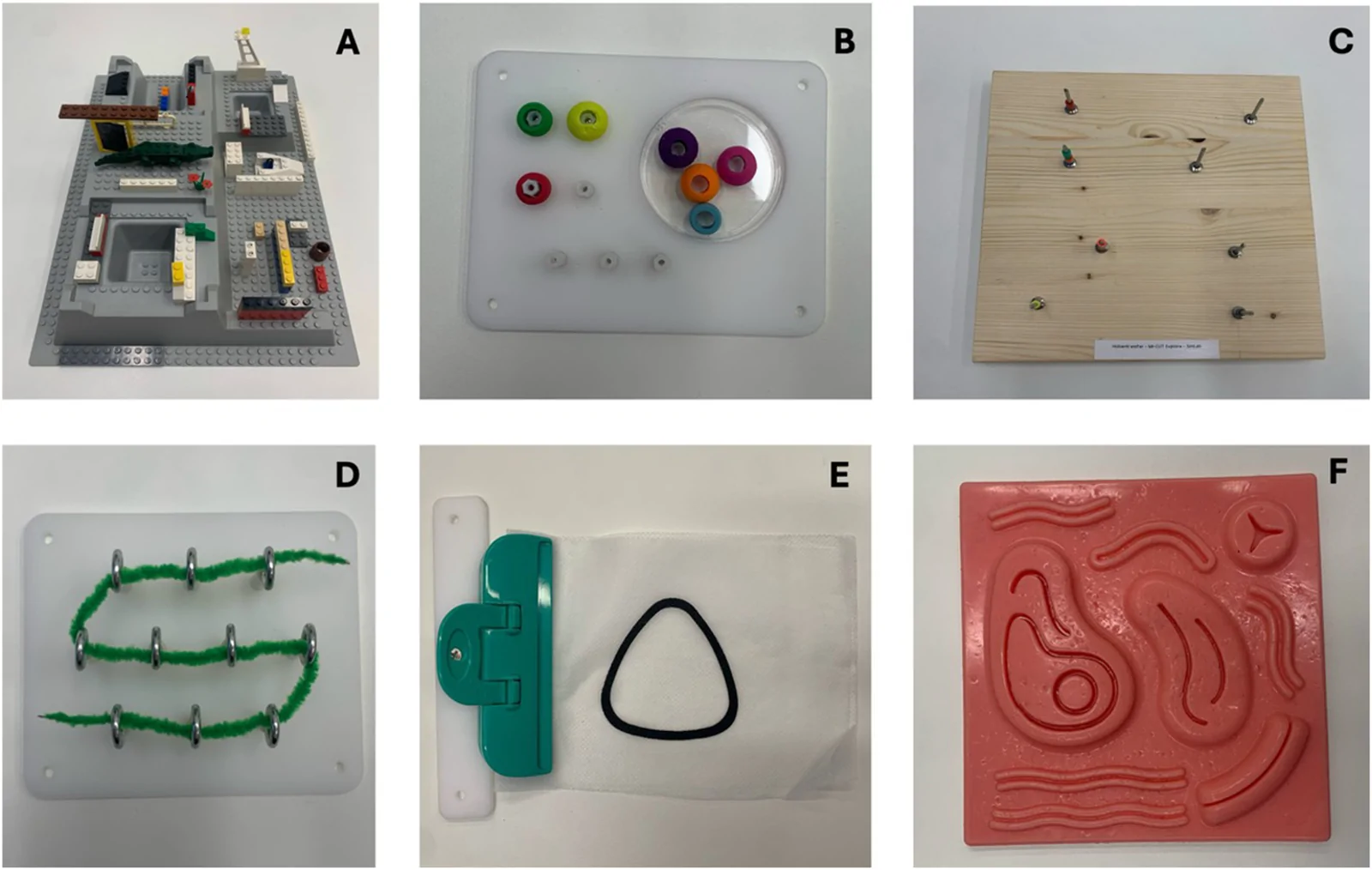
Obtained modules for the student-led peer-teaching are: Module 1:“Children’s construction model”, Module 2: “Peg-transfer Easy”, Module 3: “Peg-transfer Advanced”, Module 4: “Pipe cleaner”, Module 5, “Cutting”, Module 6: “Suturing”.

The participants reporting high levels of satisfaction (*M*=4.88/5). Nearly all students (*M*=4.98/5) indicated they would recommend the course to peers. The peer-teaching model was particularly well received, with students rating their tutors as knowledgeable and approachable (*M*=4.68/5). Participants felt less hesitant to ask questions with peer instructors than they might with physicians (*M*=4.34/5). Interest in additional instruction from faculty surgeons was relatively low (*M*=2.68/5) (Figures 1 and 2).

### Evaluation of training modules

**Figure 3:**
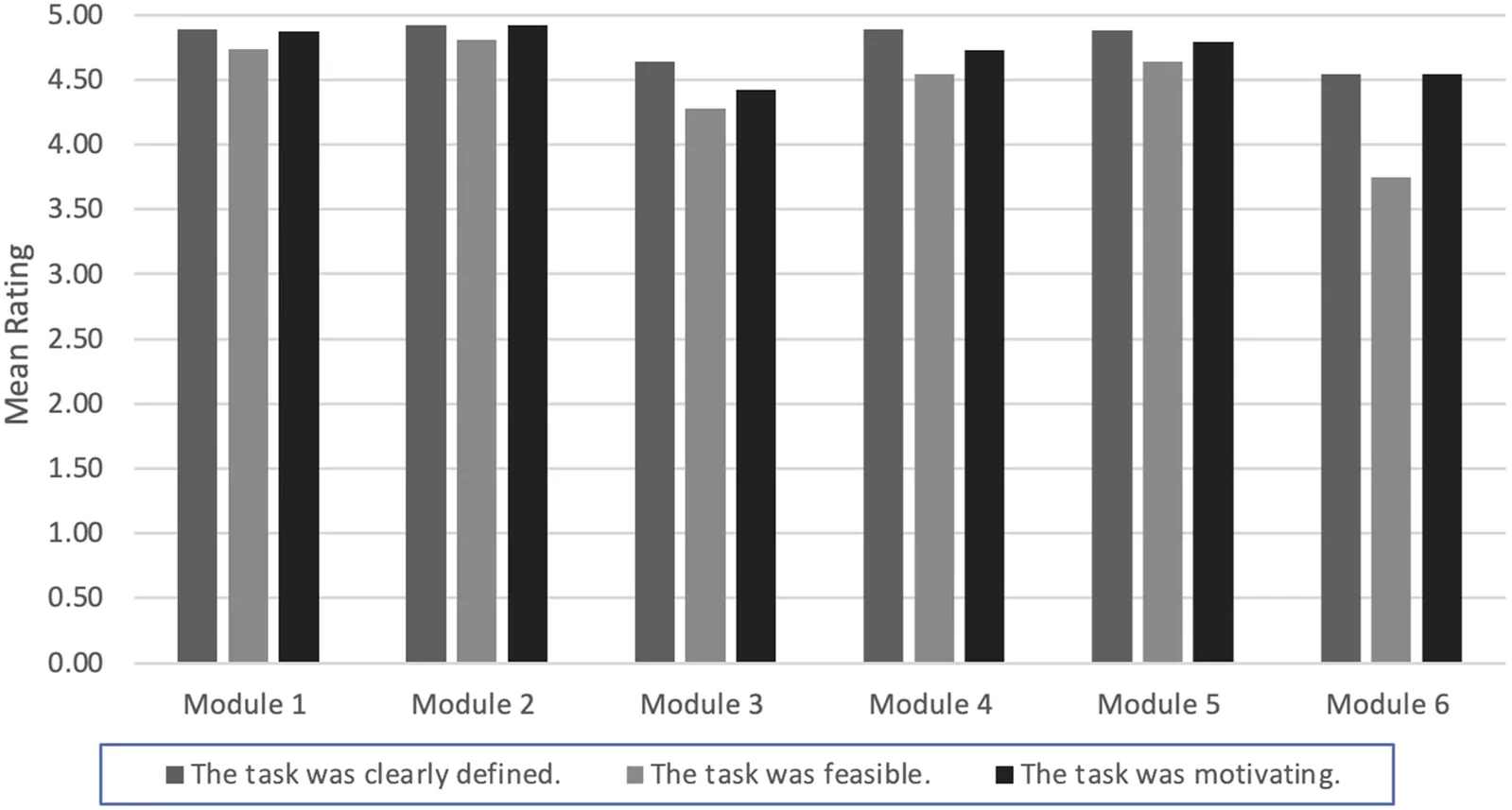
Training modules were rated using a 5-point-Likert-scale from 1=strongly disagree to 5=strongly agree. Only participants who performed the task, where able to evaluate (Module 1, “children’s construction model”, *n*=38; Module 2, “Peg-transfer Easy”, *n*=38; Module 3, “Peg-transfer Advanced”, *n*=36; Module 4, “Pipe cleaner”, *n*=37; Module 5, “Cutting”, *n*=33; Module 6, “Suturing”, *n*=24).

Each training module was assessed based on clarity, feasibility, and motivational impact. Across all modules, participants reported a high level of engagement, with the majority rating exercises as well-structured and effective for skill acquisition (Figure 3).

**Figure 4:**
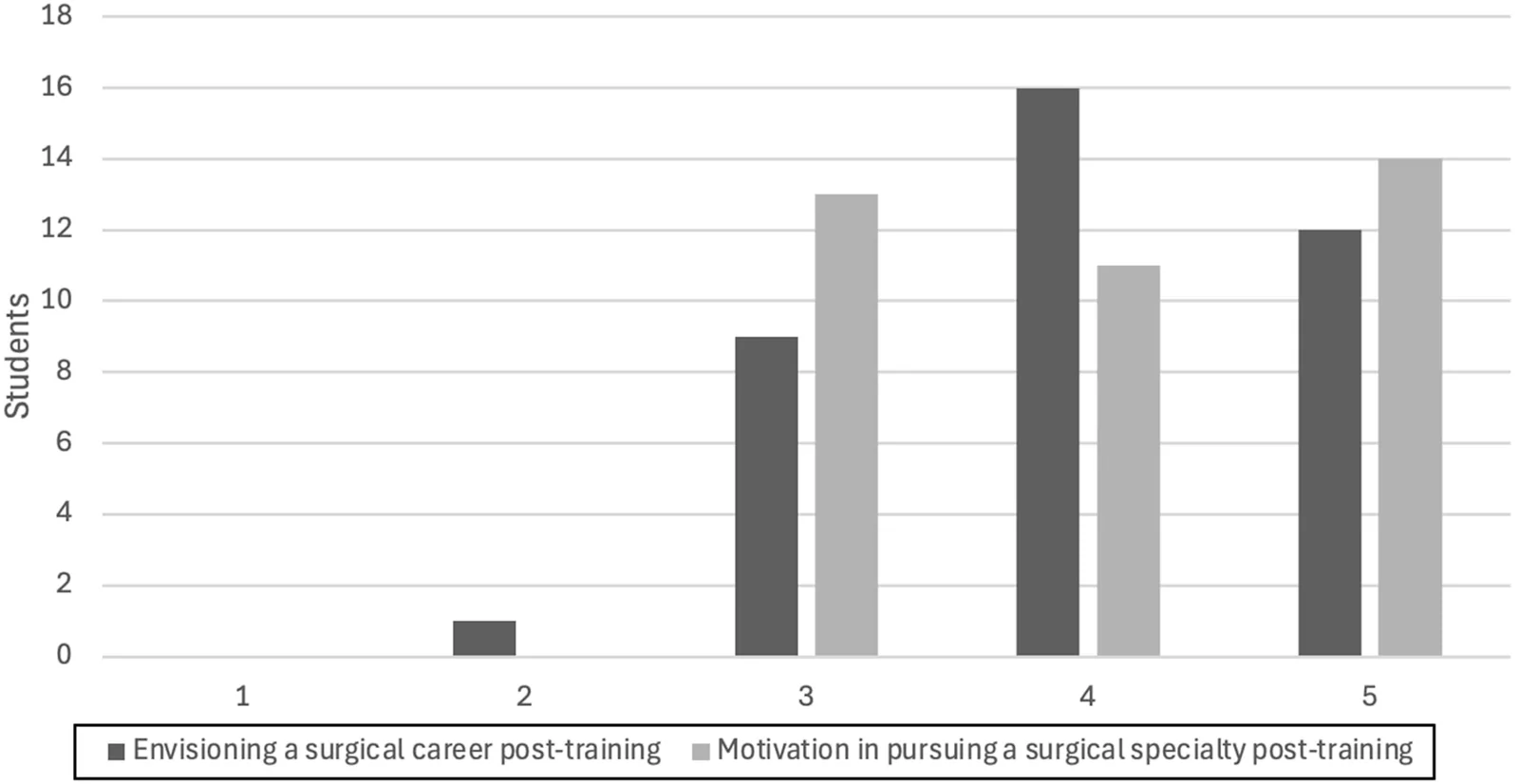
Self-perception about envisioning a surgical career and motivation in pursuing a surgical speciality was evaluates using a 5-point-likert-scale from 1=strongly disagree to 5=strongly agree.

Qualitative feedback reinforced the high satisfaction levels, with participants emphasizing the interactive and supportive learning atmosphere. Comments such as *“motivated and approachable tutors,” “fun and engaging exercises,”* and *“a great way to learn laparoscopic techniques without pressure”* were frequently noted (Figure 4).

### Impact on surgical career motivation

A total of 45 students participated in the motivational assessment (female: *n*=20, 44 %; male: *n*=25, 56 %; non-binary *n*=0, 0 %). Before training, 58 % (*n*=26) of students stated they could envision a future in surgery. The primary motivators for considering a surgical career were:

– Intrinsic passion for surgery (*n*=40, 89 %),
– Opportunities for structured skill development and further training (*n*=29, 64 %),
– Appeal of teamwork and collaborative dynamics (*n*=35, 78 %).

### Post-course evaluations demonstrated a significant increase in motivation and confidence

The majority of the participants also completed the post-test survey (*n*=38 of 45):

– 87 % (*n*=33) of participants reported feeling better prepared for clinical internships and rotations.
– 74 % (*n*=28) expressed a stronger affinity toward a surgical career.
– 50 % (*n*=19) noted an increase in motivation to actively pursue a surgical specialty.
– 66 % (*n*=25) planned to engage in further laparoscopic training.

These findings indicate that participants acquired technical skills and developed a more defined sense of professional orientation within the field of surgery.

## Discussion

This study demonstrates the effectiveness and feasibility of a student-led, peer-teaching surgical training program in fostering technical skills, surgical confidence, and professional identity formation among medical students. Unlike traditional faculty-led training, this initiative was conceptualized, developed, and executed by students, providing a unique perspective on how self-directed learning environments can complement formal medical education [15].

### Student-led peer-teaching as an effective training model

The peer-led nature of the program was a key factor in its success. Prior research has shown that student-led initiatives create engaging, accessible, and non-intimidating learning environments, allowing for interactive and effective transfer of knowledge [15], 16]. In this study, peer tutors not only taught laparoscopic techniques but also served as relatable role models, reducing hierarchical barriers and fostering an atmosphere of open communication, potentially redefining paradigms for surgical leaders [10], 17]. This structure enabled participants to feel more comfortable asking questions, making mistakes, and refining their skills without the pressure often associated with traditional faculty-led instruction [18].

Senior students, once trained by experts in the field of laparoscopic surgery, became instructors for their peers and subsequently recruited and trained new tutors, ensuring a self-sustaining and long-term continuity of the program. This system also fostered a sense of collective responsibility for surgical education, empowering students to take ownership of their learning [19], 20].

### Impact on surgical career motivation and identity formation

The Results of this study indicates that a structured, yet informal student-led training approach can significantly enhance motivation for surgical careers. Medical students often cite a lack of early exposure to hands-on surgical training as a barrier to considering surgery as a career path [8]. By providing an accessible, voluntary, and pressure-free training opportunity, this initiative enabled students to develop confidence in their technical skills, leading to a measurable increase in interest and motivation toward surgery.

Beyond technical proficiency, the program contributed to the formation of a surgical identity – a crucial factor in career decision-making [21]. In traditional surgical education, students may struggle to identify with the surgical profession due to perceived and proven stereotypes, gender biases, and work–life balance concerns [9], 22]. However, by fostering a collaborative and engaging peer-learning environment, this initiative provided participants with a sense of belonging and early professional socialization within surgery [23].

### Advantages over traditional faculty-led training

Compared to conventional faculty-led courses, student-led initiatives offer distinct advantages, including greater flexibility, lower resource dependency, and a more relatable learning environment [18], 24], 25]. Previous studies have highlighted that peer-led surgical education enhances learning outcomes by reducing intimidation, increasing participation, and fostering collaborative skill development. Additionally, students reported higher engagement, increased willingness to ask questions, and greater enjoyment of the learning process [15], 23].

Moreover, peer instructors were perceived as highly competent and effective, despite not being formally certified educators. This underscores the potential of student-driven initiatives to deliver high-quality training while achieving similar or even superior engagement and motivation, particularly when combined with structured guidance from experienced surgeons [26].

### Limitations and future considerations

Despite its successes, this study has several limitations. First, participation was voluntary, which may have resulted in a selection bias favoring students already interested in surgery [27]. However, the significant increase in motivation post-training suggests that even among a motivated cohort, the program further reinforced surgical career interest [28].

Additionally, the absence of standardized skill assessment metrics limits the ability to objectively quantify the technical proficiency gained. Future versions of this programme should integrate validated assessment tools for surgical simulations in order to more accurately evaluate skill improvement, ideally in longer formats such as a week-long course [29], [30], [31].

Furthermore, while the student-led format was a major strength of this initiative, it also presents challenges in long-term sustainability and scalability. Ensuring the consistent recruitment and training of new peer educators is essential to maintaining program quality. Future efforts should explore strategies to institutionalize student-led surgical training within medical curricula while preserving its core peer-learning principles.

## Conclusions

This study underscores the immense potential of student-led, peer-teaching surgical training programs in enhancing surgical skills, career motivation, and identity formation among medical students. By fostering a collaborative, self-sustaining, and student-driven learning environment, this initiative successfully addressed gaps in traditional surgical education without commitment of clinical financial or human resources during the courses [32].

Moving forward, medical schools and surgical departments should recognize the value of student-led training initiatives and consider incorporating them as complementary educational strategies. Empowering students to take an active role in their own surgical education may not only enhances technical skills but also cultivates the next generation of motivated and confident surgeons.

## Supplementary Material

Supplementary Material

Supplementary Material
