# Supplementary material for: Student-led curriculum design and teaching by fellow students provide a feasible platform for surgical identity and a gateway to surgical careers
Source: Innov Surg Sci. 2025 Aug 5;11(3):141–9. doi: 10.1515/iss-2025-0014 (PMC13591107; doi:10.1515/iss-2025-0014)
Supplement: Supplementary file 1 [file j_iss-2025-0014_suppl_001.pdf]

1. Socio-demographic background

1. Select your age range:
    - <18 years
    - 18-21 years
    - 22-25 years
    - 26-29 years
    - >30 years

2. Gender
    - Female
    - Male
    - Divers
    - Not specified

3. Academic year
    - Year 1
    - 2nd year
    - 3rd year
    - 4th year
    - 5th year
    - 6th year/practical year

2. Previous surgical experience

1. Have you already gained previous surgical experience?
    - Yes
    - No

2. How often have you already gained previous surgical experience?
   Indication of frequency
    - 0x
    - 1x
    - 2-5x
    - 6-10x
    - More than 10x

3. How often have you performed surgical procedures independently, e.g. suture closure in the operating theatre?
    - 0x
    - 1x
    - 2-5x
    - 6-10x
    - More than 10x

4. How often have you assisted with surgical procedures?
    - 0x
    - 1x

- 2-5x
- 6-10x
- More than 10x

5. How often have you watched surgical procedures being performed?
   - 0x
   - 1x
   - 2-5x
   - 6-10x
   - More than 10x

3. Previous laparoscopic experience

1. Have you already had previous laparoscopic experience?
   - Yes
   - No

2. How often have you had previous laparoscopic experience?
   Indication of frequency
   - 0x
   - 1x
   - 2-5x
   - 6-10x
   - More than 10x

3. How often have you already performed laparoscopic procedures, e.g. camera assistance?
   - 0x
   - 1x
   - 2-5x
   - 6-10x
   - More than 10x

4. How often have you assisted with laparoscopic procedures?
   - 0x
   - 1x
   - 2-5x
   - 6-10x
   - More than 10x

5. How often have you watched laparoscopic procedures being performed?
   - 0x
   - 1x
   - 2-5x
   - 6-10x
   - More than 10x

4. Gaming

1. Do you play video games?
    - Yes
    - No

2. How often do you play video games per week?
    - 0x
    - 1x
    - 2-5x
    - 6-10x
    - More than 10x

5. Instrument

1. Do you play an instrument regularly?
    - Yes
    - No

2. How often do you play your instrument each week?
    - 0x
    - 1x
    - 2-5x
    - 6-10x
    - More than 10x

6. Motivation

Why did you decide to do the laparoscopy training?
Multiple selection possible!

- I would like to become a surgeon.
- I would like to see more hands-on training in studies.
- I was curious and wanted to try it out.
- Other (enter as free text): _______

7. Overall satisfaction

1. Overall, I am satisfied with the laparoscopy training. (1 = strongly disagree, 3 = neutral, to 5 = strongly agree)
    - 1
    - 2
    - 3
    - 4
    - 5

2. I am interested in taking part in further laparoscopy training sessions. (1 = strongly disagree, 3 = neutral, to 5 = strongly agree)
    - 1

- 2
- 3
- 4
- 5

3. By participating in the laparoscopy training, I feel better prepared for practical work in surgery regarding the upcoming clinical traineeships. (1 = strongly disagree, 3 = neutral, to 5 = strongly agree)
   - 1
   - 2
   - 3
   - 4
   - 5

4. I would recommend participation in the laparoscopy training to my friends. (1 = strongly disagree, 3 = neutral, to 5 = strongly agree)
   - 1
   - 2
   - 3
   - 4
   - 5

8. Peer teaching

1. Overall, I am satisfied with the peer teaching. (1 = strongly disagree, 3 = neutral, to 5 = strongly agree)
   - 1
   - 2
   - 3
   - 4
   - 5

2. I am interested in taking part in further training courses that are taught in a peer-teaching format. (1 = strongly disagree, 3 = neutral, to 5 = strongly agree)
   - 1
   - 2
   - 3
   - 4
   - 5

3. I had the feeling that the peer teachers had sufficient prior knowledge and surgical skills to teach these to me. (1 = strongly disagree, 3 = neutral, to 5 = strongly agree)
   - 1
   - 2
   - 3
   - 4

- 5

4. The peer teaching format gave me the confidence to ask questions that I would not have asked medical professionals, e.g. physicians. (1 = strongly disagree, 3 = neutral, to 5 = strongly agree)
   - 1
   - 2
   - 3
   - 4
   - 5

5. I would have liked to be taught by medical professionals, e.g. physicians. (1 = strongly disagree, 3 = neutral, to 5 = strongly agree)
   - 1
   - 2
   - 3
   - 4
   - 5

6. I received exactly as much support as I needed. (1 = strongly disagree, 3 = neutral, to 5 = strongly agree)
   - 1
   - 2
   - 3
   - 4
   - 5

7. I would have liked more structured feedback from the peer teachers. (1 = strongly disagree, 3 = neutral, to 5 = strongly agree)
   - 1
   - 2
   - 3
   - 4
   - 5

9. Modules

1. Have you completed Module 1 "Children's construction model"?
   - Yes
   - No

2. Have you completed module 2 " Peg-transfer Easy"?
   - Yes
   - No

3. Have you completed module 3 " Peg-transfer Advanced"?
   - Yes
   - No

4. Have you completed module 4 "Pipe cleaner"?
   - Yes
   - No

5. Have you completed module 5 "Cutting"?
   - Yes
   - No

6. Have you completed module 6 "Suturing"?
   - Yes
   - No

10. Module evaluation (collected individually for each module)

Rate module "1" on a scale (1 = strongly disagree, 3 = neutral, to 5 = strongly agree) for the different questions.

1. The task was clearly defined.
   - 1
   - 2
   - 3
   - 4
   - 5

2. I knew what was required of me at all times.
   - 1
   - 2
   - 3
   - 4
   - 5

3. The task was realizable for me.
   - 1
   - 2
   - 3
   - 4
   - 5

4. The task motivated me and I enjoyed it.
   - 1
   - 2
   - 3
   - 4
   - 5

5. The design of the module was appropriate for the task.
   - 1

- 2
- 3
- 4
- 5

6. Stress level

1. How would you rate your stress level in relation to the laparoscopy training before teaching? (low = 1, rather low = 2, neutral = 3, rather high = 4, high = 5)
   - 1
   - 2
   - 3
   - 4
   - 5

2. What was your actual stress level during the laparoscopy training? (low = 1, rather low = 2, neutral = 3, rather high = 4, high = 5)
   - 1
   - 2
   - 3
   - 4
   - 5

3. How satisfied are you with your own performance in training? (1 = dissatisfied, 3= neutral, 5 = very satisfied)
   - 1
   - 2
   - 3
   - 4
   - 5

4. I have fulfilled my own standards and expectations of my performance in training. (1 = strongly disagree, 3 = neutral, to 5 = strongly agree)
   - 1
   - 2
   - 3
   - 4
   - 5

7. Free text comments

1. Criticism of peer teaching:
   We look forward to your feedback so that we can improve!

   ____

2. We also appreciate your positive feedback. What did you like?

____

3. We would like to hear what you would like to see at future events of this kind!

____
