# Supplementary material for: Student-led curriculum design and teaching by fellow students provide a feasible platform for surgical identity and a gateway to surgical careers
Source: Innov Surg Sci. 2025 Aug 5;11(3):141–9. doi: 10.1515/iss-2025-0014 (PMC13591107; doi:10.1515/iss-2025-0014)
Supplement: Supplementary file 2 [file j_iss-2025-0014_suppl_002.pdf]

Pretest evaluation

1. Socio-demographic background

1. Select your age range:
   - <18 years
   - 18-21 years
   - 22-25 years
   - 26-29 years
   - >30 years

2. Gender
   - Female
   - Male
   - Divers
   - Not specified

3. Academic year
   - Year 1
   - 2nd year
   - 3rd year
   - 4th year
   - 5th year
   - 6th year/practical year

2. Previous surgical experience

1. Have you already gained previous surgical experience?
   - Yes
   - No

2. How often have you already gained previous surgical experience?
   Indication of frequency
   - 0x
   - 1x
   - 2-5x
   - 6-10x
   - More than 10x

3. How often have you performed surgical procedures independently, e.g. suture closure in the operating theatre?
   - 0x
   - 1x
   - 2-5x
   - 6-10x
   - More than 10x

4. How often have you assisted with surgical procedures?

- 0x
- 1x
- 2-5x
- 6-10x
- More than 10x

5. How often have you watched surgical procedures being performed?
   - 0x
   - 1x
   - 2-5x
   - 6-10x
   - More than 10x

3. Previous laparoscopic experience

1. Have you already had previous laparoscopic experience?
   - Yes
   - No

2. How often have you had previous laparoscopic experience?
   Indication of frequency
   - 0x
   - 1x
   - 2-5x
   - 6-10x
   - More than 10x

3. How often have you already performed laparoscopic procedures, e.g. camera assistance?
   - 0x
   - 1x
   - 2-5x
   - 6-10x
   - More than 10x

4. How often have you assisted with laparoscopic procedures?
   - 0x
   - 1x
   - 2-5x
   - 6-10x
   - More than 10x

5. How often have you watched laparoscopic procedures being performed?
   - 0x
   - 1x
   - 2-5x
   - 6-10x
   - More than 10x

4. Gaming

1. Do you play video games?
   - Yes
   - No

2. How often do you play video games per week?
   Specification per week
   - 0x
   - 1x
   - 2-5x
   - 6-10x
   - More than 10x

5. Instrument

1. Do you play an instrument regularly?
   - 0x
   - 1x
   - 2-5x
   - 6-10x
   - More than 10x

2. How often do you play your instrument each week?
   Specification per week
   - 0x
   - 1x
   - 2-5x
   - 6-10x
   - More than 10x

6. Investigation of the factors influencing the motivation to work in a surgical specialty

1. Would you like to work in a surgical specialty later? (1 = not at all, 3 = neutral, to 5 = absolutely)
   - 1
   - 2
   - 3
   - 4
   - 5

2. How strongly is your motivation to work in a surgical specialty influenced by your intrinsic motivation (personal passion)? (1 = not at all, 3 = neutral, to 5 = absolutely)
   - 1
   - 2

- 3
- 4
- 5

3. How strongly is your motivation to work in a surgical specialty influenced by your manual dexterity? (1 = not at all, 3 = neutral, to 5 = absolutely)
   - 1
   - 2
   - 3
   - 4
   - 5

4. How strongly is your motivation to work in a surgical specialty influenced by your stress resilience? (1 = not at all, 3 = neutral, to 5 = absolutely)
   - 1
   - 2
   - 3
   - 4
   - 5

5. How strongly is your motivation to work in a surgical specialty influenced by your emotional stability? (1 = not at all, 3 = neutral, to 5 = absolutely)
   - 1
   - 2
   - 3
   - 4
   - 5

6. How strongly is your motivation to work in a surgical specialty influenced by the teaching at your university? (1 = not at all, 3 = neutral, to 5 = absolutely)
   - 1
   - 2
   - 3
   - 4
   - 5

7. How strongly is your motivation to work in a surgical subject influenced by extracurricular teaching programmes, e.g. surgical simulation? (1 = not at all, 3 = neutral, to 5 = absolutely)
   - 1
   - 2
   - 3
   - 4
   - 5

8. How strongly is your motivation to work in a surgical specialty influenced by mentors and role models? (1 = not at all, 3 = neutral, to 5 = absolutely)
   - 1

   - 2
   - 3
   - 4
   - 5

9. How strongly is your motivation to work in a surgical specialty influenced by career opportunities? (1 = not at all, 3 = neutral, to 5 = absolutely)
   - 1
   - 2
   - 3
   - 4
   - 5

10. How strongly is your motivation to work in a surgical specialty influenced by the salary? (1 = not at all, 3 = neutral, to 5 = absolutely)
    - 1
    - 2
    - 3
    - 4
    - 5

11. How strongly is your motivation to work in a surgical specialty influenced by your work-life balance? (1 = not at all, 3 = neutral, to 5 = absolutely)
    - 1
    - 2
    - 3
    - 4
    - 5

12. How strongly is your motivation to work in a surgical specialty influenced by hierarchies? (1 = not at all, 3 = neutral, to 5 = absolutely)
    - 1
    - 2
    - 3
    - 4
    - 5

13. How strongly is your motivation to work in a surgical specialty influenced by the behavior and tone of the team? (1 = not at all, 3 = neutral, to 5 = absolutely)
    - 1
    - 2
    - 3
    - 4
    - 5

14. How strongly is your motivation to work in a surgical specialty influenced by the use of modern technologies? ((1 = not at all, 3 = neutral, to 5 = absolutely)

- 1
- 2
- 3
- 4
- 5

15. How strongly is your motivation to work in a surgical specialty influenced by systematic training and further education courses? (1 = not at all, 3 = neutral, to 5 = absolutely)
    - 1
    - 2
    - 3
    - 4
    - 5

16. How strongly is your motivation to work in a surgical specialty influenced by the type of patient contact? (1 = not at all, 3 = neutral, to 5 = absolutely)
    - 1
    - 2
    - 3
    - 4
    - 5

17. How strongly is your motivation to work in a surgical specialty influenced by learning a broad spectrum? (1 = not at all, 3 = neutral, to 5 = absolutely)
    - 1
    - 2
    - 3
    - 4
    - 5

18. How strongly is your motivation to work in a surgical specialty influenced by teamwork? (1 = not at all, 3 = neutral, to 5 = absolutely)
    - 1
    - 2
    - 3
    - 4
    - 5

19. Are there other aspects that influence your motivation?
    - ____

7. Survey on motivational factors for later choice

The following factors are important to me for my future career choice:

1. Intrinsic motivation for the subject (personal passion) (1 = not at all important, 3 = neutral, 5 = very important)

- 1
- 2
- 3
- 4
- 5

2. Manual skills (1 = not important at all, 3 = neutral, 5 = very important)
    - 1
    - 2
    - 3
    - 4
    - 5

3. Stress resilience (1 = not important at all, 3 = neutral, 5 = very important)
    - 1
    - 2
    - 3
    - 4
    - 5

4. Emotional stability (1 = not important at all, 3 = neutral, 5 = very important)
    - 1
    - 2
    - 3
    - 4
    - 5

5. Mentors and role models (1 = not important at all, 3 = neutral, 5 = very important)
    - 1
    - 2
    - 3
    - 4
    - 5

6. Career opportunities (1 = not important at all, 3 = neutral, 5 = very important)
    - 1
    - 2
    - 3
    - 4
    - 5

7. Earning potential (1 = not important at all, 3 = neutral, 5 = very important)
    - 1
    - 2
    - 3
    - 4
    - 5

8. Work-life balance (1 = not important at all, 3 = neutral, 5 = very important)
   - 1
   - 2
   - 3
   - 4
   - 5

9. Hierarchies (1 = not important at all, 3 = neutral, 5 = very important)
   - 1
   - 2
   - 3
   - 4
   - 5

10. Behavior and team tone (1 = not important at all, 3 = neutral, 5 = very important)
    - 1
    - 2
    - 3
    - 4
    - 5

11. Use of modern technologies (1 = not at all important, 3 = neutral, 5 = very important)
    - 1
    - 2
    - 3
    - 4
    - 5

12. Systematic training and further education courses (1 = not at all important, 3 = neutral, 5 = very important)
    - 1
    - 2
    - 3
    - 4
    - 5

13. Patient contact (1 = not important at all, 3 = neutral, 5 = very important)
    - 1
    - 2
    - 3
    - 4
    - 5

14. Learning a broad spectrum (1 = not important at all, 3 = neutral, 5 = very important)

- ○ 1
- ○ 2
- ○ 3
- ○ 4
- ○ 5

15. Teamwork (1 = not important at all, 3 = neutral, 5 = very important)
    - ○ 1
    - ○ 2
    - ○ 3
    - ○ 4
    - ○ 5

16. Are there any other aspects that are important to you in your future career?
    - ○ ____

Post-test evaluation

8. Feedback on laparoscopy training in peer-teaching format

1. Overall, I am satisfied with the laparoscopy training. (1 = strongly disagree, 3 = neutral, 5 = strongly agree)
   - 1
   - 2
   - 3
   - 4
   - 5

2. I am interested in taking part in further courses. (1 = strongly disagree, 3 = neutral, 5 = strongly agree)
   - 1
   - 2
   - 3
   - 4
   - 5

3. I am interested in taking part in courses that go beyond the basic level. (1 = strongly disagree, 3 = neutral, 5 = strongly agree)
   - 1
   - 2
   - 3
   - 4
   - 5

4. I am interested in being able to train in simulated surgical scenarios, e.g. appendectomy and cholecystectomy using 3D printing and 3D casting. (1 = strongly disagree, 3 = neutral, 5 = strongly agree)
   - 1
   - 2
   - 3
   - 4
   - 5

5. By participating in the basic course, I feel better prepared for future surgical clinical traineeships and other practical assignments, e.g. practical year. (1 = strongly disagree, 3 = neutral, 5 = strongly agree)
   - 1
   - 2
   - 3
   - 4
   - 5

6. I would recommend participation in the laparoscopy training to my friends and colleagues. (1 = strongly disagree, 3 = neutral, 5 = strongly agree)

- 1
- 2
- 3
- 4
- 5

7. I liked the training in the peer-teaching format. (1 = strongly disagree, 3 = neutral, 5 = strongly agree)
   - 1
   - 2
   - 3
   - 4
   - 5

8. I had the feeling that the peer teachers had sufficient prior knowledge and surgical skills to teach these to me. (1 = strongly disagree, 3 = neutral, 5 = strongly agree)
   - 1
   - 2
   - 3
   - 4
   - 5

9. The peer teaching concept gave me the confidence to ask questions that I would not have asked medical professionals, e.g. physicians. (1 = strongly disagree, 3 = neutral, 5 = strongly agree)
   - 1
   - 2
   - 3
   - 4
   - 5

10. I received as much support as I needed during training. (1 = strongly disagree, 3 = neutral, 5 = strongly agree)
   - 1
   - 2
   - 3
   - 4
   - 5

11. I would have liked to be trained by medical professionals, e.g. physicians. (1 = strongly disagree, 3 = neutral, 5 = strongly agree)
   - 1
   - 2
   - 3
   - 4
   - 5

9. Self-perception of the participants

1. How would you rate your stress level before the laparoscopy training? (1 = low, 2 = rather low, 3 = neutral, 4 = rather high, 5 = high)
   - 1
   - 2
   - 3
   - 4
   - 5

2. How would you rate your stress level during the laparoscopy training? (1 = low, 2 = rather low, 3 = neutral, 4 = rather high, 5 = high)
   - 1
   - 2
   - 3
   - 4
   - 5

3. How satisfied are you with your own performance in training? (1 = not satisfied at all, 3 = neutral, 5 = very satisfied)
   - 1
   - 2
   - 3
   - 4
   - 5

4. I have fulfilled my own standards and expectations of my performance in training. (1 = not at all, 3 = neutral, 5 = absolutely)
   - 1
   - 2
   - 3
   - 4
   - 5

10. Motivation after participating in the training

1. Has the training at SimLab had an influence on your motivation to work in a surgical speciality? (1 = strong decrease, 3 = constant, 5 = strong increase)
   - 1
   - 2
   - 3
   - 4
   - 5

2. As a result of the training at SimLab, can you better imagine working in a surgical speciality later on? (1 = strongly disagree, 3 = neutral, 5 = strongly agree)
   - 1

- 2
- 3
- 4
- 5

3. The opportunity to take part in extracurricular laparoscopy training at SimLab in a peer-teaching format motivates me to take up a surgical subject. (1 = strongly disagree, 3 = neutral, 5 = strongly agree)
    - 1
    - 2
    - 3
    - 4
    - 5

11. Peer teaching on simulated organ models

The SimLab student research group would also like to offer advanced courses (e.g. for appendectomy and cholecystectomy) in the future.

Please select the correct answer for each point.

1. The prospect of training on simulated organ models from 3D printing and 3D casting motivates me to further improve my basic surgical skills. (1 = strongly disagree, 3 = neutral, 5 = strongly agree)
    - 1
    - 2
    - 3
    - 4
    - 5
2. The prospect of training on simulated organ models from 3D printing and 3D casting motivates me to attend further courses. (1 = strongly disagree, 3 = neutral, 5 = strongly agree)
    - 1
    - 2
    - 3
    - 4
    - 5
3. The prospect of training on simulated organ models from 3D printing and 3D casting motivates me to want to work in a surgical specialty. (1 = strongly disagree, 3 = neutral, 5 = strongly agree)
    - 1

- ○ 2
- ○ 3
- ○ 4
- ○ 5

1. Criticism of peer teaching:
   We look forward to your feedback so that we can improve!

   ____

2. We also appreciate your positive feedback. What did you like?

   ____

3. Wishes: We would like to hear what you would like to see at future events of this kind!

   ____
